# Design and first experimental results of an edgeless PET prototype for breast imaging: DeepBreast

**DOI:** 10.1186/s40658-026-00869-1

**Published:** 2026-04-16

**Authors:** Marta Freire, Santiago Jiménez-Serrano, Andrea Gonzalez-Montoro, Francisco B. García, José F. Toledo, Julio Barberá, Jorge Álamo, Irene Torres-Espallardo, Luis F. Vidal, Francis Loignon-Houle, Carlos de Alfonso, Sara Echegoyen, María J. Rodríguez-Álvarez, Filomeno Sánchez, Antonio J. González

**Affiliations:** 1 Instituto de Instrumentación para Imagen Molecular (I3M), Centro Mixto CSIC-Universitat Politècnica de València, Camino de Vera s/n, Valencia, 46022 Spain; 2 Oncovision, Carrer de Jeroni de Montsoriu, 92, Valencia, 46022 Spain; 3 Servicio de Medicina Nuclear, Área Clínica de Imagen Médica, Hospital Univ. y Polit. La Fe, Valencia, 46026 Spain

**Keywords:** Breast cancer, Edgeless PET, Monolithic scintillators, Neural networks, PET

## Abstract

**Background:**

Breast cancer causes the largest number of cancer-related deaths among women worldwide. With the aim of improving Positron Emission Tomography (PET) technology for accurate breast cancer diagnosis and staging, we propose a system design based on monolithic crystals with inherent Depth of Interaction (DOI) capabilities and an innovative edgeless detector ring. This approach eliminates the physical gaps between PET detectors, improving the system detection efficiency while potentially enhancing the image quality since edge effects are reduced. We have developed a dedicated breast PET system prototype (*DeepBreast*) to show the feasibility of this design. The system is composed of 14 curved LYSO monolithic scintillators of 12.5 mm thickness glued side-by-side with a high-refractive index compound. The useful transaxial and axial Field of View (FOV) of the system are 160 mm and 50 mm, respectively. A Neural Network technique was used for the *x*- and *y*- photon impact position estimation. The impact DOI and energy values were determined using the Voronoi calibration methodology. An initial experimental evaluation of the *DeepBreast* system has been performed inspired by the NEMA protocols for whole-body and small-animals PET scanners.

**Results:**

A nearly flat spatial resolution as a function of radial position was obtained, which indicates the DOI capability of the system to mitigate parallax errors. An average spatial resolution of 1.9 ± 0.1 mm, 1.9 ± 0.1 mm and 1.7 ± 0.1 mm FWHM was achieved at the center of the axial FOV for the radial, tangential, and axial directions, respectively. A maximum sensitivity value of 2% was measured at the center of the FOV. The noise equivalent count rate peak reached 15 kcps at 13.4 MBq. Moreover, percent contrast values of 27.9%, 28.8%, 56.8%, 72.5%, 87.2% and 84.2% were achieved for 4.5 mm, 6 mm, 9 mm, 12 mm, 15 mm and 20 mm cylinders of a larger dedicated IQ phantom, respectively.

**Conclusions:**

The initial experimental results demonstrate the feasibility of the *DeepBreast* as an innovative PET scanner for breast cancer imaging.

## Background

Breast cancer is the most common type of cancer and the major cause of cancer deaths among women worldwide [1]. Different imaging techniques, such as X-ray mammography, Magnetic Resonance Imaging (MRI), Computed Tomography (CT), or Positron Emission Tomography (PET), are used for the detection and assessment response to therapy of breast cancer patients [2]. X-ray mammography screening programs are routinely used, and have been proven effective [3, 4]. Nevertheless, this imaging technique has some limitations that need to be overcome, especially in providing more accurate diagnosis at early stages when the tumor size is still small (< 2 mm) and in assessing treatment efficacy [5]. PET technique using radiotracers such as ^18^F-fluorodeoxyglucose ([^18^F]FDG) has shown to be an effective imaging modality, facilitating breast cancer staging, distant-metastasis detection, prognostic prediction, and evaluation of the pathological response to treatment [6]. However, the lesion detection sensitivity of whole-body PET (WB-PET) decreases for smaller tumor sizes which limits its use in staging early breast cancers [7, 8, 9]. The significant advances done in clinical WB-PET scanners to achieve improved spatial resolution, Time of Flight (TOF) capabilities, and sensitivity could be used to improve this detection sensitivity [11, 12]. However, a complementary option to surpass this limitation is the use of breast-specific PET scanners employing high-resolution detectors placed close to the patient’s breast [8, 9,10, 13, 14, 15]. Indeed, several breast-specific PET systems have been developed, some of which are currently commercially available [16]. They can be classified into two main categories [17–18]: (i) Positron Emission Mammography (PEM), where two planar or curved detectors are placed on either side of the breast with upright or prone patient positioning [19–25]; and (ii) dedicated breast PET (dbPET) systems where the breast is imaged with a ring-shaped detector and the patient is usually in prone position [16, 26–28]. Recently, dedicated breast PET inserts for simultaneous operation with MRI scanners have also been developed [29, 30].

Regarding detector design, most of breast-specific PET scanners are based on pixelated scintillation crystals. These detectors have demonstrated to provide good energy resolution and discrete information of the 2D annihilation photon interaction position by using relatively simple mathematical algorithms. Nevertheless, the dead space between pixels reduces the effective detection area, thus slightly decreasing the system sensitivity. They can provide Depth of Interaction (DOI) information, but this typically involves additional hardware and/or more expensive manufacturing processes [31]. DOI information is relevant to mitigate parallax errors and to achieve homogenous spatial resolution across the entire image Field of View (FOV). For instance, one breast-specific PET scanner (*Elmammo*) was developed using staggered 4-layer pixelated detectors providing a discrete DOI resolution of 4.44–4.60 mm of each layer [26].

The use of monolithic crystals has been already proposed as an alternative to the pixelated ones. The *MAMMI* PET system is based on monolithic LYSO (Lu_1.8_Y_2_SiO_5_:Ce) crystals [16]. The spatial resolution in monolithic-based detectors is no longer limited by the pixel size and they inherently provide continuous DOI estimation without the use of additional components [32]. However, they present some performance degradation at the edges of the crystal (so-called edge effects), generated by the truncation of the Light Distributions (LDs) [33]. Moreover, they typically require more complex mathematical algorithms and time-consuming calibration procedures to provide accurate 3D annihilation impact position and energy compared to pixelated scintillators.

**Fig. 1:**
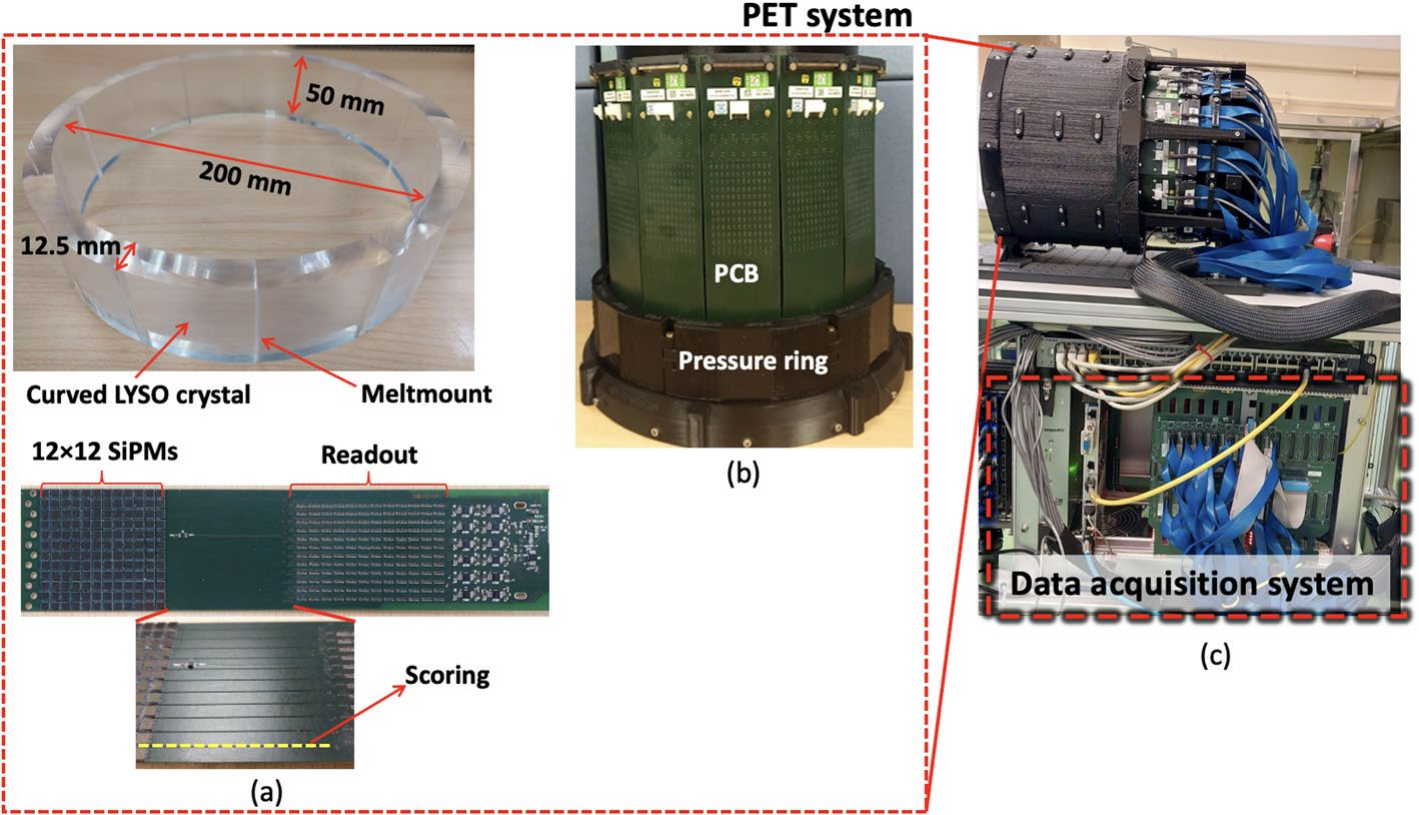
*DeepBreast* system prototype. (**a**) Scintillation ring (top) and flexible printed circuit board (bottom) containing 12 × 12 Silicon Photomultipliers (SiPMs) and the reduction readout circuitry. Scoring relates to small cuts on both sides of the PCB that allow us to bend the board without damaging the traces or coupling of the components (**b**) Assembled ring showing the PCBs and the pressure ring for robustness. The PCBs were bent when applying pressure with the pressure ring. (**c**) Photo of the *DeepBreast* system showing the data acquisition system

In this work we propose an alternative system design based on an innovative edgeless scintillation detector composed of monolithic curved LYSO crystals glued side-by-side with a high-refractive index compound, see Fig. 1(a). This approach has three main advantages: (i) it provides DOI capabilities since monolithic detectors are used, (ii) it increases the system detection efficiency since transaxial gaps are reduced and (iii) it might enhance the image quality since edge effects are mitigated [34–37]. The concept of using curved crystals instead of blocks has been previously implemented in some scanners [19, 38, 39] and the performance improvements when gluing monolithic crystals have been also demonstrated at the detector level [34, 40].

We have developed a dedicated breast PET system prototype (*DeepBreast*) as a proof of concept. In this work, we present its experimental realization and initial tests. To our knowledge, this approach has not been previously implemented in full PET systems. Furthermore, we have implemented a methodology that reduces calibration times and facilitates the overall procedure. In the following, we describe the system design and components, the 3D impact positioning methodology as well as an initial performance evaluation of the system using an assessment inspired on the National Electrical Manufacturers Association (NEMA) standards for whole-body (NEMA NU 2-2018) [41] and for small-animal (NEMA NU 4–2008) [42] PET scanners.

## Methods

### DeepBreast scanner

The *DeepBreast* PET scanner has inner and outer diameters of 200 mm and 225 mm, respectively. The system defines an axial length of 50 mm (see Fig. 1(a)) and consists of 14 curved LYSO crystals (n_LYSO_=1.8) from EPIC (Jiangsu, China). The crystal thickness is 12.5 mm, and the outer crystal face (in contact with the photosensors) has an arc length of about 50 mm [35]. The lateral sides of the crystals were optically coupled using a high-refractive-index material named Meltmount from Cargille Labs (n_Melt_=1.7) to form a single annulus (see Fig. 1(a)). Since the Meltmount is a thermoplastic material, it was heated to 70 °C to decrease its viscosity and applied to the lateral sides of the crystals to be glued. This optical coupling between crystals allows the scintillation light produced by each incident gamma ray to be shared among neighboring crystals [34]. All crystal faces were polished, and both the inner face and top and bottom faces of the ring were black painted to minimize internal reflections.

To collect the scintillation light, 14 custom Printed Circuit Boards (PCBs) each containing 12 × 12 SiPM elements (3 × 3 mm^2^ active area, 4.36 mm pitch) were used. The SiPMs were of the J-series type (35 μm cell size) from OnSemi [43]. Each PCB has 11 linear slots (scoring), see Fig. 1(a), which allows us to bend the linear slots when force is applied, see Fig. 1(b) [35]. Notice that the PCBs are only curved in the linear slot areas, but the SiPM elements are not bent. The SiPM elements are coupled to the cylindrical outer face of the crystal annulus by means of optical grease (SYLGARD 184, Dow Corning, refractive index 1.4) [44]. Each PCB includes a reduction readout circuitry (see Fig. 1(a)) that sums the SiPM output signals along each row (R) and column (C) -R&C signals-, reducing the number of signals from 124 (12 × 12) to 24 (12 + 12) [45]. Thus, the signal outputs from each PCB are the 24 R&C signals, a trigger signal (summation of the row signals) used to select coincidence events, and a temperature signal.

The Data Acquisition (DAQ) system includes 7 custom Analog to Digital Converter boards (ADCs) with 12-bit precision, a trigger card with a coincidence time window of 5 ns, and the acquisition computer, all housed in a crate, as shown in Fig. 1(c). The ADCs were programmed with a 250 ns integration window. These electronics do not provide TOF capabilities.

**Fig. 2:**
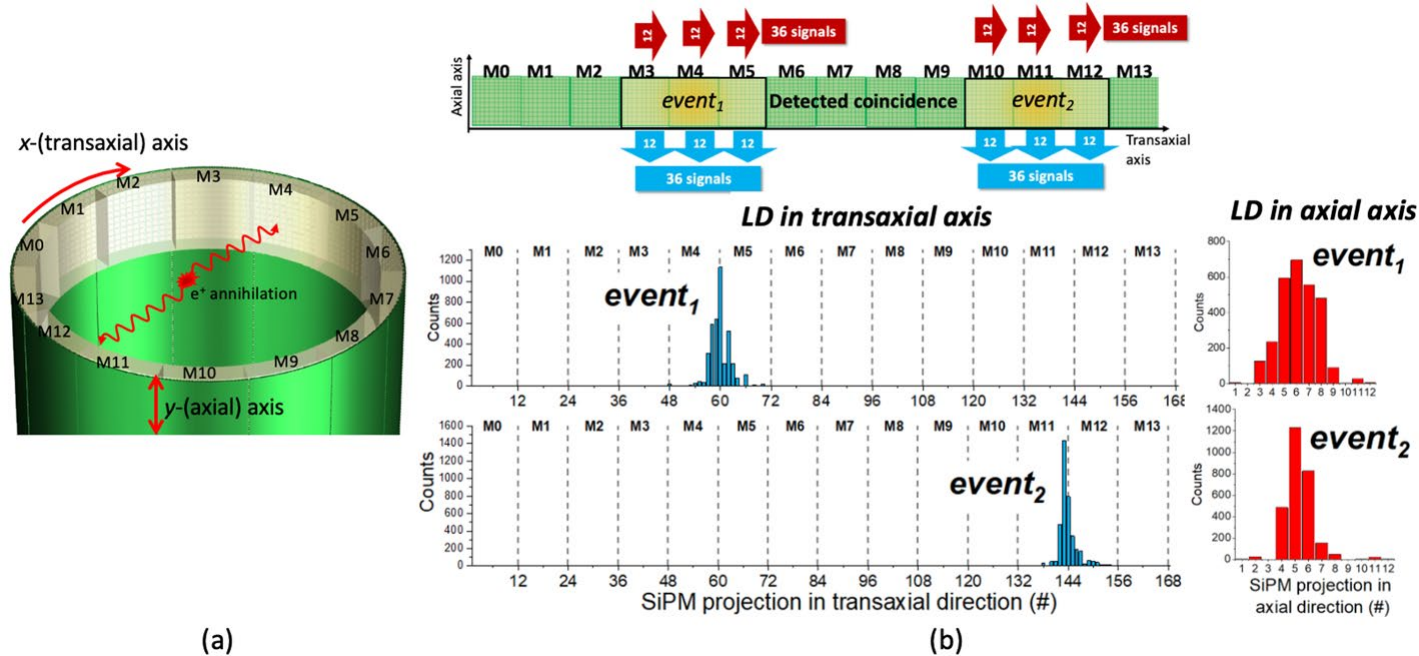
(**a**) Sketch of the *DeepBreast* system with the 14 modules labeled (M0-13). (**b**) Light distribution profiles along the *x*-(transaxial) axis and *y*-(axial) axis for the event_1_ and *event*_*2*_ of a coincidence

The signal outputs from each PCB are transferred to the DAQ by means of multicoaxial cables. The DAQ works as if the system is composed of 14 modules (M0-M13) (see Fig. 2(a)), and it allows coincidences between each module and its seven opposite ones. This defines a useful transaxial FOV of 160 mm. When a coincidence {*event*_*1*_, *event*_*2*_} is detected, the signals coming from the two involved modules plus their neighbor ones at its left and right, are sent to the acquisition computer with the same coincidence time flag. Thus, for every detected coincidence, a total of 36 + 36 row signals (*x*- transaxial) and 36 + 36 column signals (*y*- axial) are digitized by the ADCs and sent to the acquisition computer (see Fig. 2(b)). In addition to the digitized values of all R&C SiPM signals, the detection time and module ID (0–13) are also sent.

### Data processing

The signals coming from each coincidence {*event*_*1*_, *event*_*2*_} were treated as follows:

i) In the *x*-(transaxial) axis: for each event, the signals of all the 14 modules in the *x*-(transaxial) axis are added together from M0 to M13, so a total of 168 signal values are used (see Fig. 2(b) bottom).
ii) In the *y*-(axial) axis: only 12 signals are considered for each event that results from the summation of the column signals from the 3 involved modules in that direction (see Fig. 2(b) bottom).

The estimation of the 3D annihilation photon impact coordinates and energy is performed separately for *event*_*1*_ and *event*_*2*_, and the methodology is described in the following sections.

### x-(transaxial) and y-(axial) impact position calculation

**Fig. 3:**
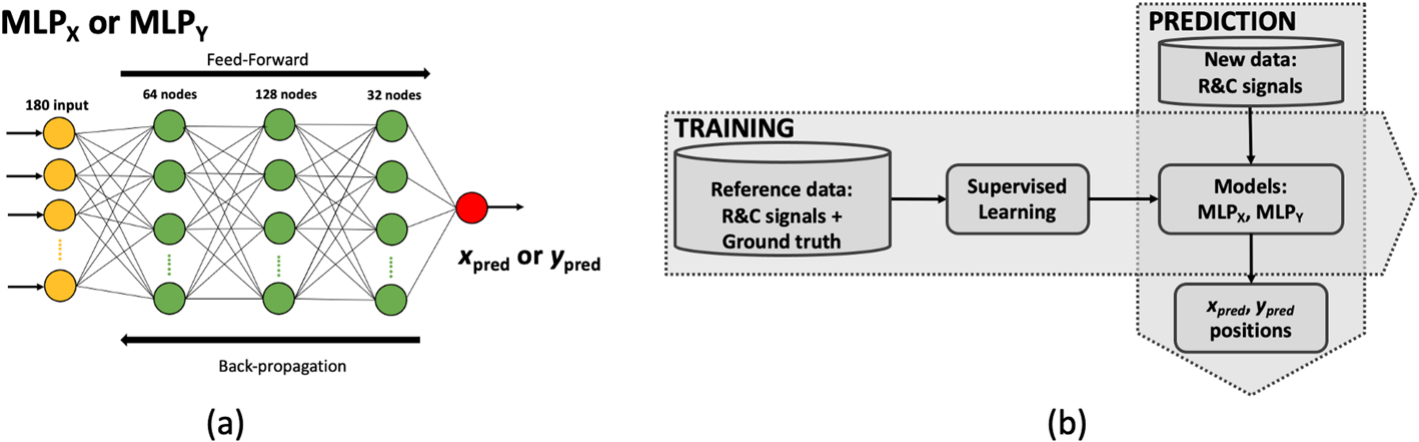
(**a**) MLPs structure used for the prediction of *x*-(transaxial) and *y*-(axial) impact coordinates. (**b**) Workflow of the training and prediction processes of the MLPs

A NN technique based on two Multilayer Perceptrons (MLPs), named MLP_X_ and MLP_Y_, is used for the estimation of the (*x*, *y*) photon impact position of coincidence events. The inputs of MLP_X_ and MLP_Y_ are the180 signals (168 signals in the *x*-(transaxial) axis and 12 signals in the *y*-(axial) axis). Both MLPs are composed of 3 layers, with 64, 128, and 32 nodes, respectively (see Fig. 3(a)).

**Fig. 4:**
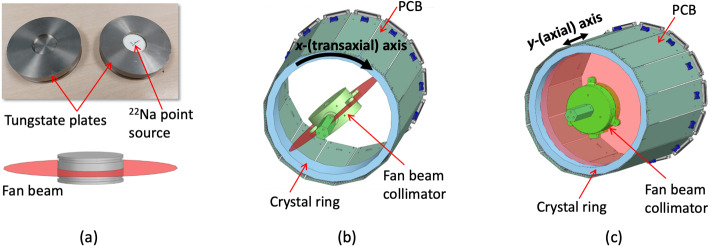
(**a**) Photographs and sketches of the fan beam collimator. (**b**) Sketch of the experimental set-up for the training of MLPx. (**c**) Sketch of the experimental set-up for the training of MLP_Y_

For the MLPs training process (see Fig. 3(b)), experimental data were acquired using a fan beam collimator composed of two tungsten collimators (76 mm in diameter and 15 mm thickness each) and a ^22^Na spherical source (0.25 mm in diameter, 320 MBq) between them [46]. The tungsten plates are mounted with a thin separation (0.4 mm), generating a fan beam irradiation (see Fig. 4(a)). This collimator design allowed us to reduce the complexity when acquiring all the reference data since it permits one to acquire all the training data with the system already mounted. For MLP_X_ training, the collimator was moved (rotated) in steps of 0.8° in the transaxial direction (see Fig. 4(b)), yielding a total of 450 measurements. For MLP_Y_ training, the collimator was linearly moved in the axial direction in steps of 0.5 mm (see Fig. 4(c)), providing 99 measurements. A total of 11k events and 200k events per calibration position were recorded in the transaxial and axial directions, respectively. For each slit position, a ± 30% energy window around the photopeak was used. Moreover, a position filter was applied using the center of gravity distribution. Events that yielded a value < 10% of the peak amplitude of the distribution were removed [34, 47]. The filtered data were randomized, and split into train (80%), evaluation (5%), and test (15%) datasets [34]. The training was performed separately for MLP_X_ and MLP_Y_ using their corresponding training dataset and Mean Absolute Error (MAE) loss function, Adam optimizer, and ReLU activation function. During the training, the MLPs were evaluated using the evaluation dataset to avoid overfitting.

The (*x*, *y*) photon impact positions of the coincidences from any new measurement were predicted using the trained MLP_X_ and MLP_Y_ (see Fig. 3(b)).

### Energy and DOI calculation

The energy of each coincidence event is estimated as the sum of the projected value of the 36 rows of SiPMs in the *x*-(transaxial) axis (see Fig. 2(b)). The DOI coordinate (defined as *z*-coordinate) was calculated as the ratio of the energy (*E*) to the maximum row value (*I*_*max*_), *E/I*_*max*_ method [48]. For the calibration process, experimental data were acquired using a non-collimated ^22^Na spherical source (0.25 mm in diameter, 0.318 MBq) placed at the cFOV. The energy and DOI were calibrated into keV and mm units, respectively, using the Voronoi calibration methodology. See reference [33] for a detailed explanation of the process. The detector surface was divided into 139 × 10 regions using the Voronoi diagrams. Then, correction factors were calculated for each region and the natural neighbor interpolation was applied to obtain two look-up-tables (LUT), namely: LUT_E_ and LUT_DOI_. For the DOI calibration, the limits of the E/I_max_ histograms for each region, named *a* and *b*, were obtained using the analytical expression for the DOI distribution [49]. A linear fit applied to these parameters (*a* and *b*) was used to calibrate the DOI values into millimeters. The parameters of the linear fit were included in the LUT_DOI_.

The mean and standard deviation of the energy and DOI resolution were computed for the 14 modules using the experimental data acquired with the ^22^Na source at the cFOV [49].

### Image reconstruction and image corrections

**Fig. 5:**
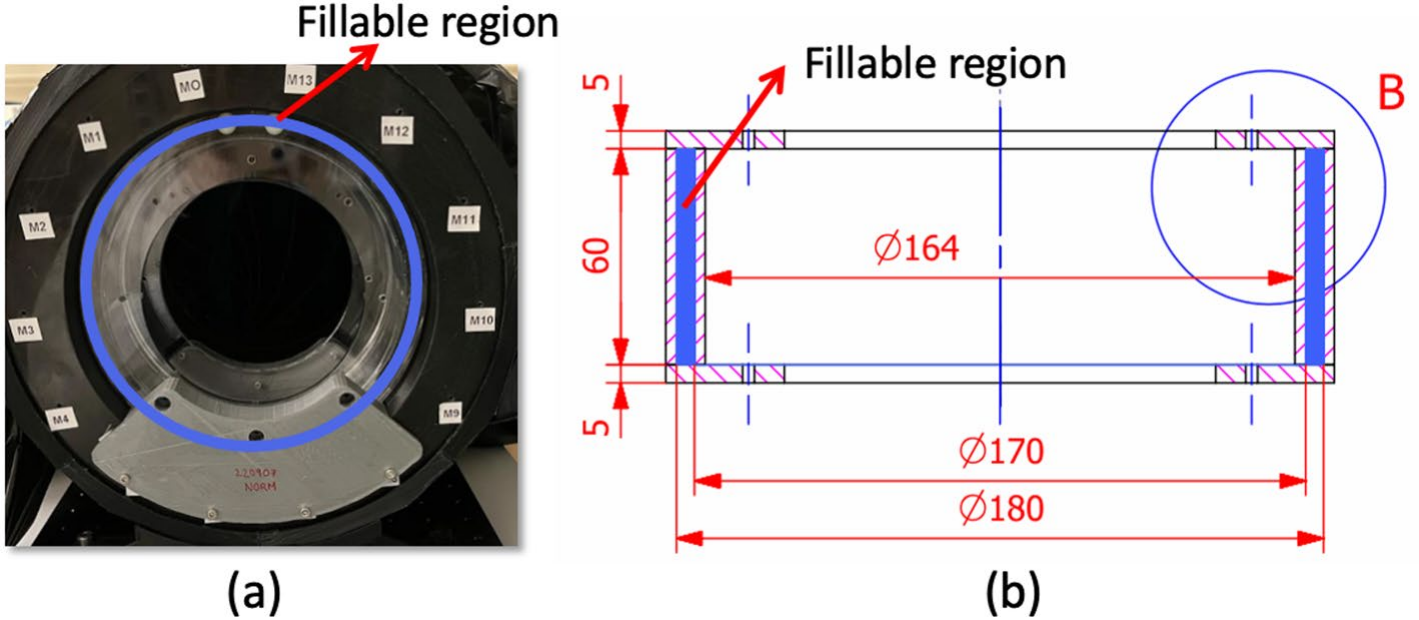
(**a**) Photograph of the normalization phantom while acquiring data. The blue line indicates where the fillable region is. (**b**) Sketch of the normalization phantom. Dimensions are in mm

3D Ordered-Subset Expectation-Maximization (OSEM) method was implemented using 0.5 × 0.5 × 0.5 mm^3^ voxel size [50, 51]. The DOI information was included to mitigate parallax errors. For this, the 3D impact position centroids of each coincidence were corrected by computing the intersections of the correct Line of Response (LOR) with the detector reference planes [52]. For the normalization, a ring phantom made of Poly(methyl methacrylate) (PMMA) material was used. The phantom contains a fillable region of 10 mm thickness at 87.5 mm off-center (see Fig. 5(b)). The ring phantom was filled with 13.9 MBq of [^18^F]FDG and located at the center of the scanner. Experimental data were acquired for 6 h (see Fig. 5(a)). From this measurement, we computed the normalization factors per LOR which were incorporated during the image reconstruction process. Scatter correction was implemented using the dual-energy window method [53]. The two energy windows were defined as 358–409 keV for scatter estimation and 408–613 keV for true coincidences. The attenuation effect was also considered by using a synthetic attenuation map based on the position of each phantom. Random, dead time and quantification corrections have not been implemented yet.

### System performance and image quality

**Fig. 6:**
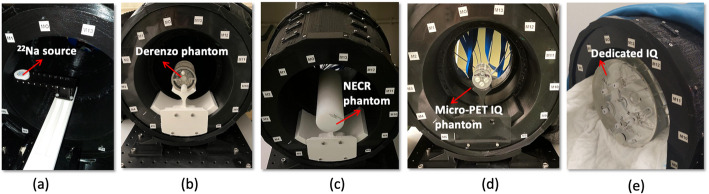
Photograph of the different measurements acquired with the *DeepBreast*. (**a**) Measurement with the ^22^Na source. (**b**) Measurements with the Derenzo phantom. (**c**) Measurements with the NECR phantom. (**d**) Measurements with the Micro-PET IQ phantom. (**e**) Measurements with the dedicated IQ

The performance of the *DeepBreast* scanner was evaluated in terms of spatial resolution, sensitivity, count rate, and image quality. The geometry of dedicated breast PET systems impedes directly using any of the available standard NEMA protocols for whole-body (NEMA NU 2-2018) [41] or small-animals (NEMA NU 4–2008) [42]. Therefore, we have followed a methodology inspired by both NEMA standards but with some deviations according to our dedicated PET system geometry. Figure 6 shows photographs of the different measurements acquired with the *DeepBreast* PET system.

### Spatial resolution

Experimental data were acquired by moving a ^22^Na spherical source (0.25 mm in diameter, 320 kBq) at different radial distances ranging from 0 mm to 80 mm, in steps of 10 mm (see Fig. 6(a)). Two sets were acquired, one with the source placed at the cFOV and the other at one-fourth of the axial FOV [42]. The images were reconstructed with OSEM method. Profiles were taken through the reconstructed images of the point source image along axial, radial, and tangential directions, and the spatial resolution values were determined by measuring the FWHM of a Gaussian fit to the profiles. The measured spatial resolution of a small size source is influenced by the number of OSEM iterations and subsets considered. Therefore, the data from the point source located at the cFOV was reconstructed for different number of iterations, from 1 to 10 (1 subset for all cases), to select the optimum iteration number to minimize artificial overestimation.

**Fig. 7:**
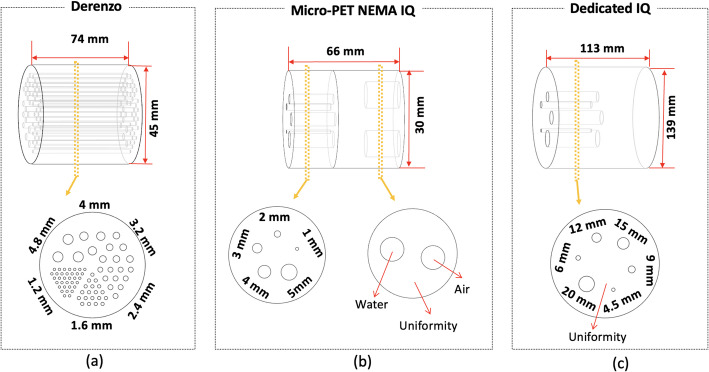
Sketches of different phantoms used in this work. (**a**) Dedicated Derenzo-like phantom. (**b**) Micro-PET NEMA IQ phantom for small animals. (**c**) Dedicated IQ phantom

Data from a custom dedicated Derenzo-like phantom were acquired to further study the image spatial resolution. The phantom is 51 mm in diameter and composed of cylindrical hot spots with diameters ranging from 1.2 to 4.8 mm (see Fig. 7(a)). The phantom was filled with [^18^F]FDG (10.4 MBq), and experimental data were acquired for 1 h (see Fig. 6(b)). In this case, the image was reconstructed using 20 iterations and 5 subsets of the OSEM method. The image was quantitatively evaluated by calculating two parameters, namely the average valley-to-peak ratio (VPR) and the resolvability of the hot spots [54]. Line profiles across the hot spots were drawn and the VPR values (VPR_spot_) estimated for each pair of two adjacent spots within a sector. The average VPR value for each sector was calculated as the mean of the VPR_spot_ values corresponding to each sector. Then, to provide a quantitative estimation of the minimum spatial separation that can be resolved by the *DeepBreast*, the average VPR values were evaluated using the Rayleigh criterion [54, 55]. This methodology has been already adopted by other groups working with hot spot phantoms [56, 57]. Following this criterion, average VPR values below 0.73 denote that the spots in the phantom sector are resolved. Additionally, the resolvability was calculated as the percentage of adjacent spot pairs belonging to the same sector with a VPR below 0.73.

### Sensitivity and count rates performance

For the sensitivity evaluation, the ^22^Na spherical source (0.25 mm in diameter, 320 kBq) was moved in steps of 2 mm across the axial direction at the center of the transaxial FOV. The sensitivity was computed for an energy window of 408–613 keV (± 20% at the 511 keV peak).

The NEMA NU 4–2008 was followed using the rat-like phantom for the count rate performance evaluation [42]. This phantom is a cylinder with a diameter of 50 mm and a length of 150 mm. The phantom includes a cylindrical drilled hole (3.2 mm diameter) parallel to the central axis at a radial distance of 17.5 mm. A fillable line source made of a flexible tubing with a diameter of 2 mm and a length of 150 mm was inserted in the drilled hole and filled with 50 MBq of [^18^F]FDG. Experimental data were acquired for 20 h in acquisitions of 480 s each (see Fig. 6(c)). The trues, random + scattered (R + S), and Noise Equivalent Count Rate (NECR) values were estimated for an energy window of 408–613 keV (± 20% at the 511 keV peak) [42].

### Image quality characterization

For the image quality characterization, the NEMA Micro-PET Image Quality (IQ) phantom was measured (see Fig. 6(d) and Fig. 7(b)) [42]. The NEMA IQ phantom is 50 mm long and 30 mm in diameter. The first half of the cylinder offers a fillable cavity of 30 mm in diameter (referred to as uniformity region) that comprises two smaller 15 mm long cavities to be filled with water and air (cold regions). The second half of the phantom contains five rods of 1, 2, 3, 4, and 5 mm in diameter. The phantom was filled with 5.3 MBq of [^18^F]FDG, and measured for 1 h (see Fig. 6(d)). The image was reconstructed using the OSEM algorithm with 6 iterations and 5 subsets. To quantitatively analyze the NEMA IQ phantom, the uniformity, spill-over ratios (SORs), and Recovery Coefficients (RCs) were calculated as follows:

i. The uniformity values were obtained by drawing a cylindrical Volume of Interest (VOI) of 22.5 mm diameter and 10 mm long over the center of the uniform region of the IQ phantom. Then, the mean (Mean_background_) and standard deviation (STD_background_) of the activity in this VOI were calculated.
ii. The RCs were estimated by drawing five circular VOIs around the hot rods of the IQ phantom, such that the hot rods were centered in the VOI. Each VOI had a diameter that is twice the rod diameter and a length of 10 mm. The voxels along the axial axis were averaged, and the maximum average activity inside each VOI (Maximum_VOI_) was determined. The RC was calculated for each hot rod as the ratio between the Maximum_VOI_ and the Mean_background_. Additionally, the standard deviation of each RC was calculated as the standard deviation of the activity along the axial axis at the position of the maximum average activity.
iii. The SORs were determined by drawing two cylindrical VOIs with a diameter of 4 mm and a length of 7.5 mm inside the center of the two cold cylinders of the IQ phantom. The SORs are defined as the ratio of the mean activity inside these VOIs and the Mean_background_.

In addition to these tests, a custom-built phantom (dedicated IQ) was also measured to further evaluate the image quality (see Fig. 6(e)). The specific dedicated IQ is 139 mm in diameter and 113 mm in height (see Fig. 7(c)). It is composed of a warm background (BG) compartment with six fillable cylindrical inserts with inner diameters of 4.5, 6, 9, 12, 15, and 20 mm. The six inserts (50 mm long each) are placed 30 mm away from the transaxial center of the phantom. The inserts and background were filled with [^18^F]FDG to have a cylinder-to-background concentration ratio (concentration_insert_/concentration_background_) equal to 5:1. The concentration of the warm background was 7.19 kBq/ml and the acquisition time was 45 min. The image was reconstructed using OSEM with 5 subsets and different number of iterations ranging from 1 to 8. For the image quality evaluation, six cylindrical Regions of Interest (ROIs) with diameters matching each insert were drawn on a slice centered along the insert. The mean hot spot values (*C*_*H, j*_) were then measured for each ROI. Six additional ROIs for each hot spot size (with same diameter as spot) were drawn and distributed along the uniform warm area at that slice of the phantom, then at four other axial positions (30 ROIs in total for each size). The measured mean values were averaged to obtain the background level for each cylinder (*C*_*B, j*_). Then, the percent contrast for each insert (*Q*_*H, j*_) and the percent background variability was obtained for each iteration number according to the NEMA protocol for whole-body (NEMA NU 2-2018) [41].

## Results

### Energy and DOI resolution

An average energy resolution of 19 ± 2% and an average DOI resolution of 3.6 ± 0.1 mm was measured at the detector level for all 14 modules.

### Spatial resolution

**Fig. 8:**
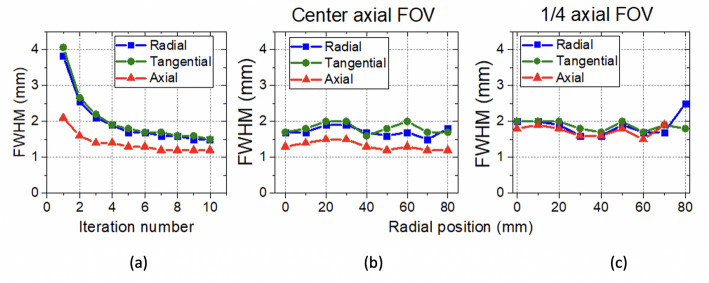
(**a**) Spatial resolution values (including DOI information) as a function of the number of iterations for the ^22^Na point source located at cFOV. (**b**) Spatial resolution values (including DOI information) at several radial positions at the center of the axial FOV. (**c**) Spatial resolution values (including DOI information) at several radial positions at one-fourth of the axial FOV

Figure 8(a) shows the reconstructed spatial resolution (FWHM) as a function of the number of iterations. Since the spatial resolution in the axial direction first converges at 7 iterations, this number was used for the different radial positions acquired. Figure 8(b) and (c) report the radial, tangential, and axial components of the reconstructed spatial resolution (FWHM) as a function of the source radial position at the center of the axial FOV and at one-fourth of the axial FOV, respectively. Spatial resolution values of 1.7 mm, 1.7 mm, and 1.3 mm FWHM were achieved at the cFOV for radial, tangential, and axial directions. In general, the dispersion of the spatial resolution for each component (radial, tangential and axial) along the different radial positions is at most 10%. We have obtained average and standard deviation values of 1.9 ± 0.1 mm, 1.9 ± 0.1 mm and 1.7 ± 0.1 mm FWHM for the radial, tangential, and axial directions, respectively, considering all measured sources at the center of the axial FOV; and 1.9 ± 0.1 mm, 2.0 ± 0.1 mm and 1.6 ± 0.1 mm FWHM for all the sources at one-fourth of the axial FOV.

**Fig. 9:**
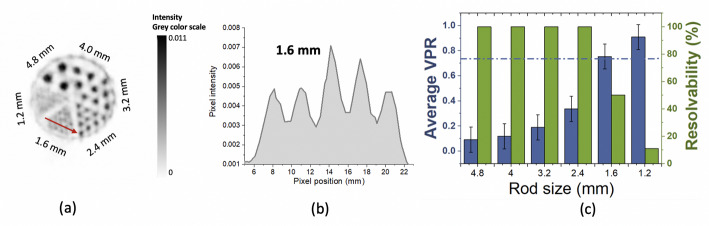
(**a**) Transaxial view of the reconstructed image of the Derenzo-like phantom. The image was obtained by summing over 8 slices. (**b**) Profile along 5 selected capillaries of 1.6 mm marked with an orange arrow in the central panel. (**c**) Average VPR (in blue) and resolvability (in green) values for each sector of hot spots of the Derenzo-like phantom

Figure 6(b), shows a photograph of the Derenzo-like phantom inside the *DeepBreast* system. Figure 9(a) and (b) provide the reconstructed image of this phantom and a profile across some of the 1.6 mm capillaries, respectively. Figure 9(c) presents the average VPR and resolvability values obtained for the hot spot sectors. Following the Rayleigh criterion, it can be observed that all the hot spots from 4.8 to 2.4 mm can be resolved.

### Sensitivity and count rates performance

**Fig. 10:**
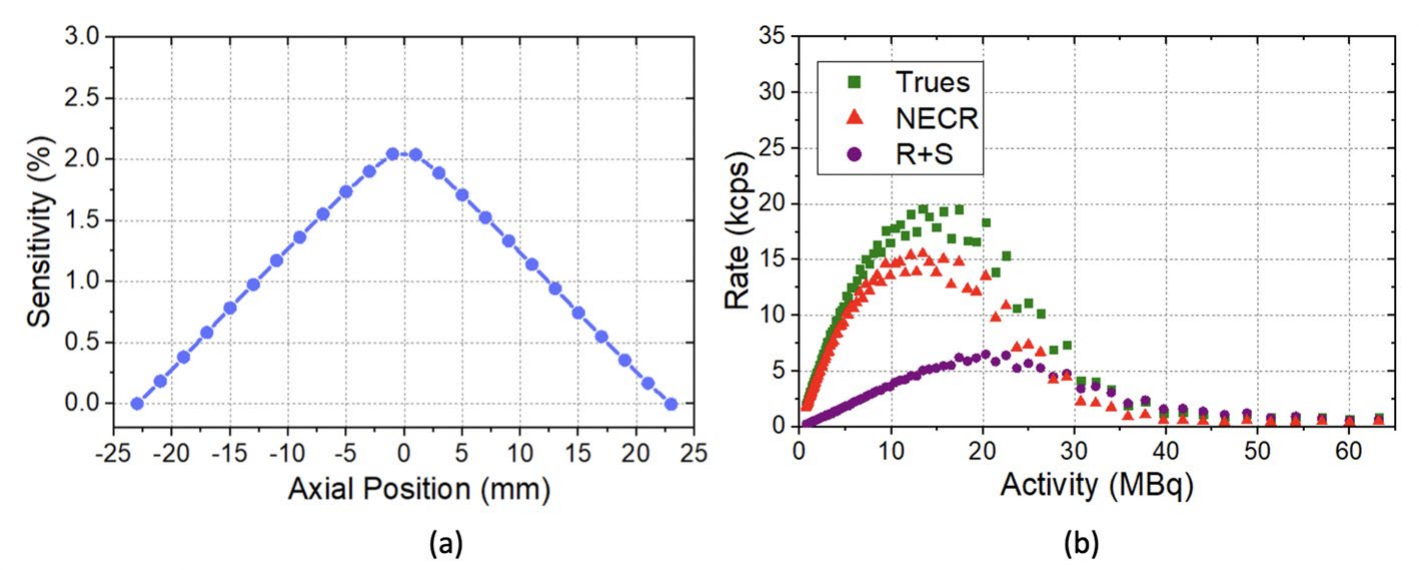
(**a**) Sensitivity values obtained for the 408–613 keV energy window (20% energy window). (**b**) Trues, R + S, and NECR curves for the 408–613 keV energy range (20% energy window) as a function of the activity in the phantom

The sensitivity curve measured with the ^22^Na source is shown in Fig. 10(a) for an energy window of 408–613 keV. At the cFOV, the peak sensitivity calculated for this energy window was 2.0%.

The trues, random plus scattered (R + S), and NECR values obtained for the 408–613 keV energy range as a function of the activity in the rat-like phantom are depicted in Fig. 10(b). A maximum NECR of 15 kcps at 13.4 MBq was obtained from these curves.

### Image quality characterization

**Fig. 11:**
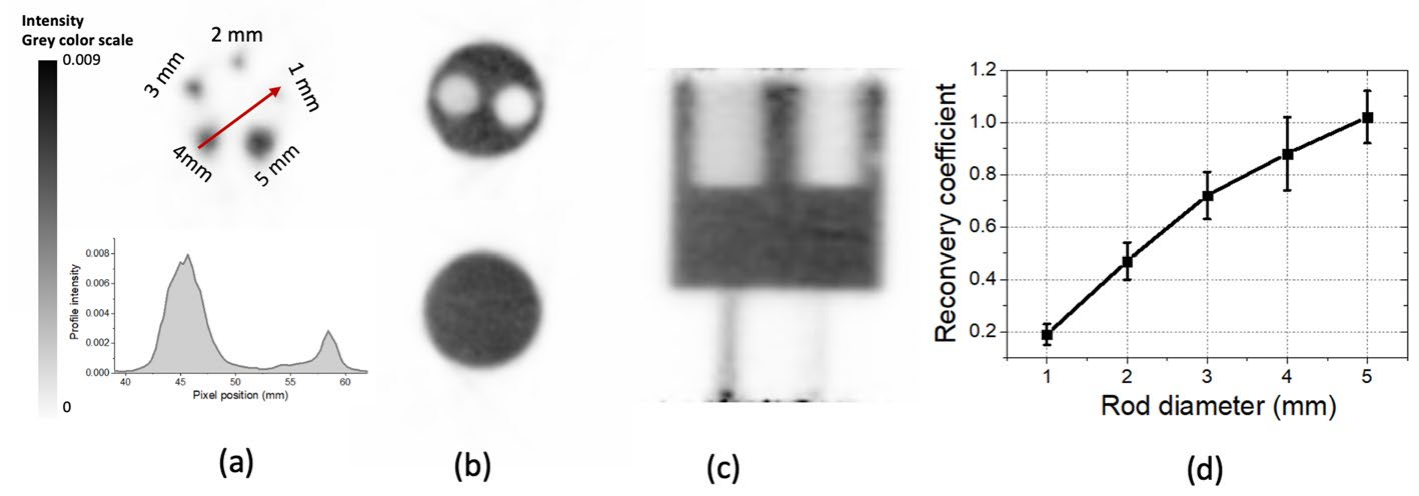
(**a**) Transaxial view of the reconstructed image for the rod region chamber of the Micro-PET NEMA IQ phantom and profile along the 4 mm and 1 mm rods. (**b**) Transaxial view of the air-water region chamber (top) and uniformity region chamber (bottom) of the Micro-PET NEMA IQ phantom. (**b**) Coronal view of the reconstructed image of the Micro-PET NEMA IQ phantom. (**b**) Recovery Coefficients for each rod

The reconstructed images of the NEMA Micro-PET IQ phantom (depicted in Fig. 7(b)) are shown in Fig. 11. The RC values and their standard deviation (error bars) as a function of the rod diameter are depicted in Fig. 11(d). A uniformity value (STD_background_) of 5% was determined. and SOR values of 6% and 23% were obtained for air and water regions, respectively.

**Fig. 12:**
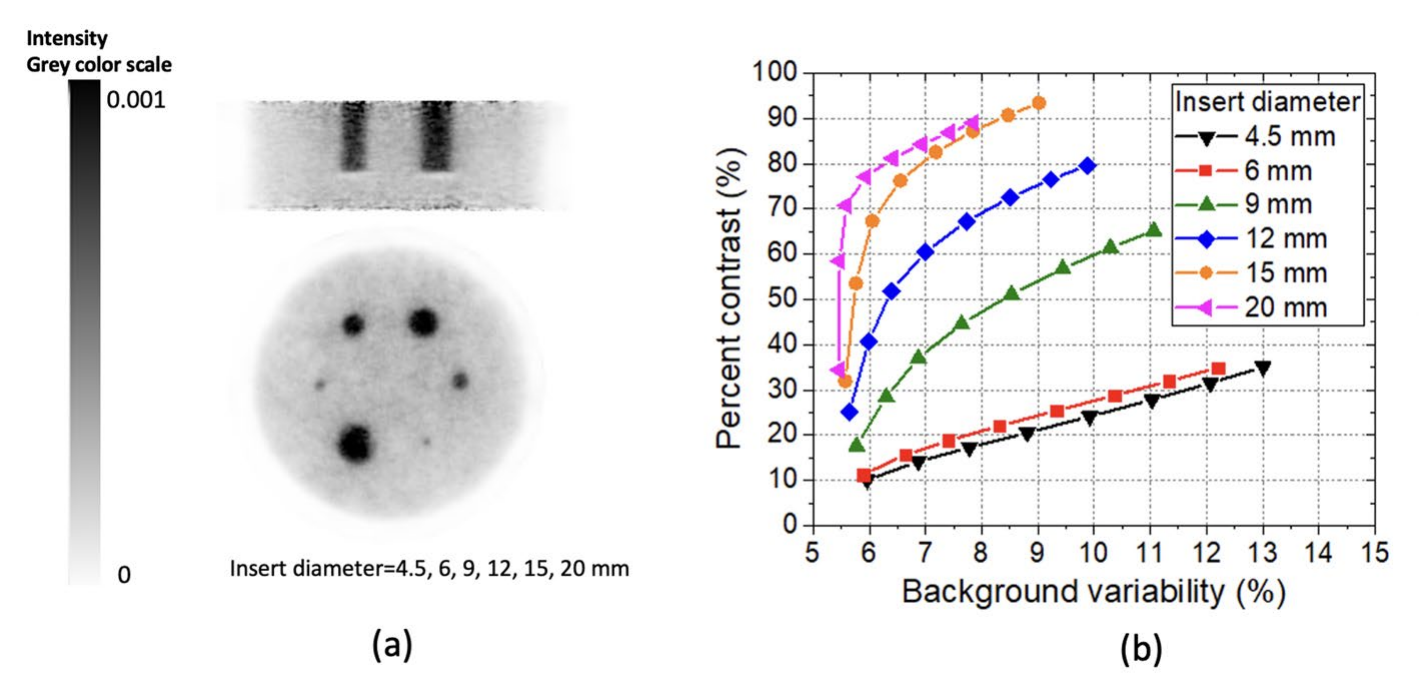
(**a**) Transaxial and coronal views of the reconstructed images of the dedicated IQ phantom. (**b**) Percent contrast values versus background variability calculated for each insert diameter and for 1–8 iterations with 5 subsets

The reconstructed images of the dedicated IQ phantom (5:1 cylinder-to-background concentration ratio) using 6 iterations and 5 subsets are shown in Fig. 12(a). All the inserts are well distinguished. Figure 12(b) shows the percent contrast values versus the background variability for each insert, where each point corresponds to an iteration number (ranging from 1 to 8, with 5 subsets in all cases).

The percent contrast and background variability values for 6 iterations and 5 subsets are shown in Table 1.

**Table 1:** Percent contrast and background variability for the dedicated IQ phantom using 6 iterations and 5 subsets

Spot size (mm)	20	15	12	9	6	4.5
Percent contrast (%)	84.2	87.2	72.5	56.8	28.8	27.9
Background variability (%)	6.9	7.8	8.5	9.4	10.4	11.1

## Discussion

We have designed and developed a novel dedicated breast PET system prototype called *DeepBreast.* The scanner is based on 14 curved LYSO monolithic crystals laterally glued using a high-refractive-index material (Meltmount). In a previous work we proposed a single LYSO scintillation tube for a small-animal PET system [59], but this approach could not be applied to the *DeepBreast* because of limitations in the scintillation crystal growth process of LYSO [60].

The proposed system design allows improving the detector energy and spatial resolutions by mitigating the edge effects related to monolithic PET detectors [34]. However, one of the main drawbacks of using these detectors in PET systems is the complexity and time-consuming calibration procedures that are usually required [32]. In this work, we have implemented an approach that reduces calibration times and facilitates the process because it allows us to obtain all the required data when the system is already mounted. For the NN training along the transaxial and axial directions, the proposed set-up allows us to reduce the acquisition time from 11 days (if experimental data are individually obtained for each detector before mounting them on the system) to 3.6 days. In addition, we have measured an average DOI resolution of 3.6 ± 0.1 mm after applying a simple Voronoi-based methodology. Similar DOI resolution values could be achieved with pixelated crystal configurations, but this would require additional hardware and/or higher manufacturing cost [31]. The energy resolution performance could be improved by using reflective coatings on the crystal sides instead of black paint. In this study, we decided to use black paint to minimize undesirable reflections in the scintillator sides.

An initial experimental evaluation of the *DeepBreast* system has been performed using an assessment that combines two NEMA protocols: whole-body (NEMA NU 2–2018) [41] and small-animal (NEMA NU 4–2008) [42] PET scanners. Table 2 compares the spatial resolution, sensitivity and NECR peak for some ring-shaped dedicated breast systems, including the *DeepBreast*. Note that this comparison is not straightforward since a large variety of methods and protocols have been used in each case. Nevertheless, we show this table to provide an indicative overview of the performance reported by the different systems.

Regarding spatial resolution, data were acquired using a ^22^Na point source and reconstructed using OSEM algorithm. Since spatial resolution values have been calculated using an iterative method, some overestimation might remain as no warm background was added [62]. Nevertheless, a homogenous spatial resolution was found across the useful FOV (see Fig. 8(b)) since parallax errors have been corrected by including the continuous DOI information before image reconstruction. The good and nearly uniform spatial resolution is expected to enhance the ability to detect very small breast tumors in the entire FOV as well as low uptake ones, which would be hardly detectable using conventional WB-PET scanners [7]. Compared to the *Mammi* system, which also uses an iterative reconstruction method, the *DeepBreast* system shows an improved spatial resolution, as the measured values are a slightly better overall and also remain constant across the useful FOV (see Table 2). The spatial capabilities were also evaluated using a Derenzo-like phantom. Some non-uniformities are visible in the reconstructed image, especially in the active line surrounding the rods (see Fig. 9(a)). We guess this could be related to dead time and random effects, which are currently not corrected.

The edgeless design of the *DeepBreast* system eliminates the transaxial gaps present in modular-based PET designs, thus enhancing the detection efficiency. An improvement of about 21% compared to a traditional design with 5 mm gaps was estimated using theoretical calculations [58]. The measured sensitivity value of 2.0% is slightly better than other dedicated-breast PET scanners with similar FOV (*BRPET* and *MAMMI* systems) (see Table 2).

Regarding the count rate performance (see Fig. 10(b)), the NECR curve is approximately linear for activities lower than ∼7 MBq and worsens from ∼20 MBq mainly due to the saturation of the electronics in terms of data transferring. The activity value reached at the NECR peak is lower in comparison with other breast-dedicated scanners with similar FOV (*BRPET* and *Mammi* systems) mostly due to our trigger configuration (see Table 2). However, we feel confident this should not compromise the studies to be conducted because, based on the images of breast PET scans already acquired with the *Mammi* system using [^18^F]FDG, the expected activity in the affected breast varies from 3.7 to 7.4 MBq for typical injected activities ranging from 180 to 370 MBq [61]. Therefore, considering that the NECR curve is nearly linear for activities lower than ∼7 MBq in the *DeepBreast*, this ensures that the system would, most likely, perform accurately during a breast scan.

**Table 2:** Performance parameters for some breast dedicated PET (ring-shape), including the *DeepBreast* system

System	Detector type	DOI	FOV (mm)	Spatial resolution (mm)	Sensitivity (%)	NECR Peak	Ref.
*PEMi*	LYSO pixelated	No	110–128	2.2–4.2^**^	6.88	37.2 MBq	[27]
*MAMMI*	LYSO monolithic	Yes	170−40	1.8–7.0^***^	1.6	44 MBq	[16]
*Elmammo*	LGSO pixelated	Yes	183−155	1.6–2.7^*^	11.2	24.9 MBq	[26]
*BRPET*	LYSO pixelated	No	205*–49	1.7-3.0^*^	0.97	21 MBq	[28]
*DeepBreast*	Curved LYSO monolithic	Yes	160−50	1.7–1.8^****^	2.0	13.4 MBq	

The table shows the spatial resolution along the radial direction, sensitivity at the center FOV and NECR peak values. Two values for the FOV are shown: the first value is the transaxial FOV and the second the axial FOV. Two FWHM values for the spatial resolution are shown: the first value is at the center of the radial direction and the second is at radial position far from the center. *Aperture, **FBP, ***MLEM, **** OSEM

Regarding image quality, the measured RC values increase with the rod diameter and the SOR value for the water-filled chamber is higher than for the air-filled chamber, as expected. Similar RC values are obtained compared to *Elmammo* and *PEMi* systems [26, 27]. The image quality was further evaluated using the larger dedicated phantom (139 mm in diameter). Some background activity is observed at the edges of the useful transaxial FOV (160 mm), most likely due to some angular undersampling.

Initial results confirm the feasibility of the prototype; however, this first design is a proof of concept and still presents some limitations. Further developments are necessary to overcome these challenges and enable its clinical translation. In particular, increasing the axial length of the system would further enhance system sensitivity and allow complete breast coverage. Moreover, in the present evaluation, reconstructed images of the phantoms were corrected by attenuation effects using a synthetic attenuation map. In future works we will consider applying attenuation correction by means of PET image segmentation [63]. In addition, random, dead time and quantification corrections would need to be implemented [64].

TOF imaging is now routinely implemented in clinical PET systems. However, for our system (∼ 200 mm in diameter) we estimate that a Coincidence Time Resolution (CTR) of ∼ 100 ps would be required to achieve a substantial effect in the Signal to Noise Ratio for lesions of ∼ 2 mm [65]. Although some recent studies have demonstrated CTR values approaching 100 ps using novel detector designs [66], further efforts are still needed both in detector technology and in system-integration.

## Conclusions

We have developed the first proof-of-concept PET dedicated to breast imaging following the proposed edgeless design, aiming to enhance sensitivity while minimizing edge effects at the detectors. The system comprises 14 curved LYSO monolithic crystals glued side-by-side using a high refractive index compound. A novel calibration methodology has been applied to accurately determine the 3D photon impact positions and energy while reducing data acquisition and simplifying the procedure. This is particularly relevant as it mitigates one of the main limitations of monolithic detectors, thereby, demonstrating its potential for dedicated systems. This methodology could be extended to standard monolithic or semi-monolithic crystal-based designs.

The first experimental evaluation of the *DeepBreast* indicates that the proposed edgeless design could be suitable for breast cancer imaging, especially due to its high DOI capabilities that allows us to reach a uniform radial spatial resolution of 1.7–1.8 mm across the entire useful FOV. Corrections for random events and dead time will be required to improve the system performance and provide quantitative PET images.

## Data Availability

The data supporting the results of this study are available upon reasonable request to the corresponding author.

## Abbreviations

ADC: Analog to Digital Converter
cFOV: Center of the Field of View
CT: Computed Tomography
DAQ: Data Acquisition
dbPET: Dedicated breast PET
DOI: Depth of Interaction
FDG: Fluorodeoxyglucose
FOV: Field of View
FWHM: Full Width at Half Maximum
IQ: Image Quality
LD: Light Distribution
LOR: Line of Response
LUT: Look-up-table
LYSO: Lutetium–Yttrium Oxyorthosilicate
MAE: Mean Absolute Error
MLP: Multilayer Perceptrons
MRI: Magnetic Resonance Imaging
NECR: Noise Equivalent Count Rate
NEMA: National Electrical Manufacturers Association
NN: Neural Network
OSEM: Ordered-Subset Expectation-Maximization
PCBs: Printed Circuit Boards
PEM: Positron Emission Mammography
PET: Positron Emission Tomography
PMMA: Poly(methyl methacrylate)
RC: Recovery Coefficients
SiPM: Silicon Photomultiplier
TB-PET: Total Body-PET
TOF: Time of Flight
VOI: Volume of Interest
VPR: Valley-to-peak Ratio
WB-PET: Whole Body-PET
